# Development of sustainable research excellence with a global perspective on infectious diseases: Centre de Recherches Médicales de Lambaréné (CERMEL), Gabon

**DOI:** 10.1007/s00508-020-01794-8

**Published:** 2021-01-04

**Authors:** Michael Ramharter, Selidji T. Agnandji, Ayôla A. Adegnika, Bertrand Lell, Ghyslain Mombo-Ngoma, Martin P. Grobusch, Matthew McCall, Riko Muranaka, Andrea Kreidenweiss, Thirumalaisamy P. Velavan, Meral Esen, Frieder Schaumburg, Abraham Alabi, Christiane Druml, Benjamin Mordmüller, Carsten Köhler, Peter G. Kremsner

**Affiliations:** 1 Department of Tropical Medicine, Bernhard Nocht Institute for Tropical Medicine & I. Dep. of Medicine University Medical Center Hamburg-Eppendorf, Hamburg, Germany; 2 Centre de Recherches Médicale de Lambaréné, Lambaréné, Gabon; 3 German Center for Infection Research, Hamburg-Lübeck-Borstel-Riems, Germany; 4 Institute of Tropical Medicine, Travel Medicine and Human Parasitology, University Clinics, Eberhard Karls University Tübingen, Tübingen, Germany; 5 German Center for Infection Research, Tübingen, Germany; 6 Department of Parasitology, Leiden University Medical Center, Leiden, The Netherlands; 7 Medical University of Vienna, Vienna, Austria; 8 Faculty of Medicine, University of Libreville, Libreville, Gabon; 9 Center of Tropical Medicine and Travel Medicine, Amsterdam University Medical Centers, University of Amsterdam, Amsterdam, The Netherlands; 10 Institut für Medizinische Mikrobiologie, Universitätsklinikum Münster, Münster, Germany

**Keywords:** Medical research, Sub-Saharan Africa, Gabon, Capacity development, Neglected tropical diseases

## Abstract

Medical research in sub-Saharan Africa is of high priority for societies to respond adequately to local health needs. Often enough it remains a challenge to build up capacity in infrastructure and human resources to highest international standards and to sustain this over mid-term to long-term periods due to difficulties in obtaining long-term institutional core funding, attracting highly qualified scientists for medical research and coping with ever changing structural and political environments. The Centre de Recherches Médicales de Lambaréné (CERMEL) serves as model for how to overcome such challenges and to continuously increase its impact on medical care in Central Africa and beyond. Starting off as a research annex to the Albert Schweitzer Hospital in Lambaréné, Gabon, it has since then expanded its activities to academic and regulatory clinical trials for drugs, vaccines and diagnostics in the field of malaria, tuberculosis, and a wide range of poverty related and neglected tropical infectious diseases. Advancing bioethics in medical research in Africa and steadily improving its global networks and infrastructures, CERMEL serves as a reference centre for several international consortia. In close collaboration with national authorities, CERMEL has become one of the main training hubs for medical research in Central Africa. It is hoped that CERMEL and its leitmotiv “to improve medical care for local populations” will serve as an inspiration to other institutions in sub-Saharan Africa to further increase African capacity to advance medicine.

Since its foundation, the Centre de Recherches Médicales de Lambaréné (CERMEL) has made a remarkable change from a research department of a small non-governmental hospital in semi-rural Gabon to one of the leading research institutions for tropical medicine in Central Africa. For institutions striving to contribute sustainably to people’s health in sub-Saharan Africa, CERMEL may serve as a role model. Here, the institutional history of CERMEL is presented with landmark activities and challenges in the quickly evolving environment of clinical research in sub-Saharan Africa.

In 1913, the first hospital in Lambaréné was founded by Albert Schweitzer. Schweitzer, the 1952 Nobel Peace Prize laureate, early on expressed his belief that routine medical practice in Africa had to be accompanied by medical research. It was in the 1970s that a new hospital complex was constructed after Schweitzer’s death, and it was only in 1981 that the first research department was founded as an annex to the modern Albert Schweitzer Hospital (Fig. 1). During that period, a collaboration with the Bernhard Nocht Institute for Tropical Medicine in Germany, was established to perform first epidemiological surveys in the central region of Gabon.

**Fig. 1:**
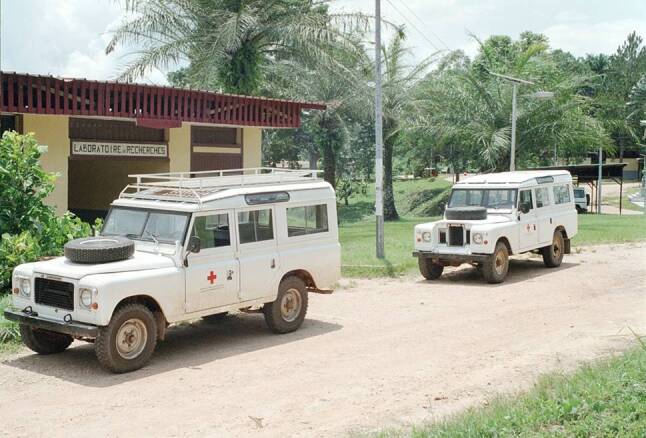
First building of the research unit at the campus of the Albert Schweitzer Hospital. (© Michael Ramharter)

In 1992 Peter G. Kremsner, who had been affiliated with the Institute for Tropical Medicine in Berlin and later became Professor for Tropical Medicine, Travel Medicine and Human Parasitology at the University of Tübingen, Germany, was appointed Scientific Director of the International Foundation of the Albert Schweitzer Hospital. A new building with modern laboratories was inaugurated in 2006, which considerably boosted improved clinical and laboratory research capacity. Increases in projects, staff and funding made the department internationally recognized with an impact beyond the hospital’s healthcare activities (Fig. 2). In 2011, this research department finally became legally independent, and became the Centre de Recherches Médicales de Lambaréné (CERMEL). Since then, CERMEL has kept on expanding its scope by participating in internationally funded research programs and became an international medical research hub with more than 200 national and international staff members. Main funding sources are the European Union (EU), especially with its European Developing Countries Clinical Trials Partnership (EDTCP), the German Federal Ministry of Education and Research, prominently via the German Center for Infection Research and the Bill and Melinda Gates Foundation. Besides these funding organizations, several mid-term to long-term research programs were conducted with funding from international drug developers and non-profit organizations such as Medicines for Malaria Venture (MMV). In addition, strong Gabonese national support was given by provision of generous infrastructure.

**Fig. 2:**
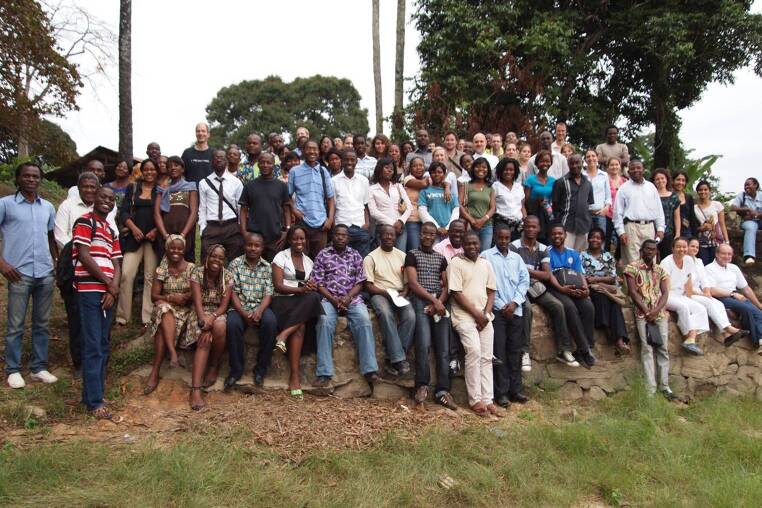
International team of researchers at the CERMEL. (© Bertrand Lell)

## Antimalarial drug trials and combination therapies

The first and most recognized activity of CERMEL is the clinical development of antimalarial drugs and antimalarial combination therapies in light of the threat of increasing drug resistance of *Plasmodium falciparum* malaria in Central Africa. Over the past three decades, CERMEL was engaged in the clinical development of most antimalarial drugs currently marketed and in clinical use in the world today.

CERMEL was the first to report chloroquine resistance in Gabon in 1984 [1]. This finding later led to the evaluation of clindamycin monotherapy and combination therapies of clindamycin-chloroquine and clindamycin-quinine for the treatment of chloroquine-resistant falciparum malaria [2–7]. Atovaquone-proguanil [8–10], arteflene [11] and sulfadoxine/pyrimethamine [12] were developed and evaluated for therapeutic as well as prophylactic use. CERMEL was also among the first African centres to develop artemisinin-based combination therapies, the current international standard for malaria chemotherapy. Those studies included the very first clinical trial of artesunate-amodiaquine [13], as well as artesunate-clindamycin [14], artesunate-mefloquine [13], fixed-dose artesunate-pyronaridine [15] and artesunate-fosmidomycin [16] as well as comparison of several of those [17] for uncomplicated *P. falciparum* malaria in African children. The first randomized trial of tafenoquine prophylaxis for *P. falciparum* malaria in the endemic area was also conducted by CERMEL [18]. Based on these first trials and subsequent drug development programs, tafenoquine became registered for malaria prevention and for radical cure of *P. vivax* infections [19].

In parallel, several new antimalarial compounds were developed as single or combination therapy in phase I–II clinical trials, such as fosmidomycin [20–22], clindamycin-fosmidomycin, [16, 23–27], piperaquine-fosmidomycin [28], chlorproguanil-dapsone [29], ferroquine and ferroquine-artesunate combinations [30–32]. In recent years, the compounds with new modes of action, such as artefenomel, ganaplacide and cipargamin [33] were tested at CERMEL. At the same time, single-dose therapies for malaria to improve patient adherence to therapeutic regimens were evaluated [33, 34]. Importantly, this clinical research was complemented by series of trials assessing simplified regimens for parenteral artesunate in severe malaria [35, 36].

As antimalarial drug development in vulnerable populations became a further public health priority [37], trials using quinine monotherapy for pregnant African women [38], and sulfadoxine-pyrimethamine as prophylactic treatment in infants and mefloquine in pregnant women [39–43] were conducted as priorities. This rich research portfolio made CERMEL globally known as a leading centre for antimalarial drug development focussing on the needs of African populations.

## Vaccine trials for malaria and other infectious diseases

In addition to the antimalarial drug development, CERMEL is dedicated to the development of new vaccines. The first vaccine trial conducted at CERMEL was the assessment of vaccines against typhoid fever and cholera [44]. Partnering with 10 research centers in sub-Saharan Africa, CERMEL participated in phase II trials and the pivotal phase III trial to assess the safety, immunogenicity and efficacy of the RTS,S/AS01 malaria vaccine, which later became the first marketable vaccine against malaria and a human parasitic disease of any kind recommended by the European Medicines Agency in 2015 [45]. Since then, several other malarial vaccine candidates including whole attenuated *P. falciparum* sporozoite vaccine candidates (PfSPZ) have been assessed in multiple clinical trials [46–49].

With the growing research capacity, vaccine trials for diseases other than malaria started to be conducted at CERMEL. One highlight was the first phase I clinical trial of the Ebola surface glycoprotein vaccine vectored with the recombinant vesicular stomatitis virus (rVSV∆G-ZEBOV-GP). The good safety and immunogenicity data from this trial supported the decision to conduct a phase III trial in the West African countries affected by the Ebola outbreak of 2014–2016, and which led to the availability of the first Ebola vaccine in experimental use in the Western and Central African outbreaks in 2018–2019 [50–57]. The rVSV∆G-ZEBOV-GP vaccine became the world’s first approved Ebola vaccine by the European Medicines Agency (Ervebo®^)^ in November 2019. Further clinical trials to characterize immune and molecular signatures by rVSV∆G-ZEBOV-GP in pediatric and adult populations are being conducted.

Other fields of vaccine development included the evaluation of recombinant Na-APR‑1 (M74) and Na-GST‑1 hookworm vaccine candidates as well as postmarketing trials assessing the impact of helminths on immunogenicity of vaccines for influenza, meningitis and cholera [58–62].

## Diagnostic studies for malaria and schistosomiasis

Thick blood smear microscopy, still the gold standard in malaria testing endorsed by WHO, was optimized in the early days of the CERMEL by describing the so-called Lambaréné method [63]. With this method quick and accurate quantification of malaria parasite densities in a patient’s blood is possible, even under basic laboratory conditions. In the Severe Malaria in African Children (SMAC) network of which CERMEL was a founding member, a series of studies were performed to identify risk factors of pediatric mortality in Africa. This is the largest study of severe malaria in history [64–66]. In the SMAC network new treatment regimens of parenteral artesunate were established for severe malaria [35, 36]. For better identification of children at highest risk of death from severe malaria, the Lambaréné organ dysfunction score (LODS) was developed [67], which is a simple scoring tool to predict the prognosis based only on three clinical factors, which are coma, prostration and deep breathing.

With funds from the European Developing Countries Clinical trial Partnership and German Center for Infection Research (DZIF), a series of *Schistosoma* antigen-based tests are being systematically studied in pregnant women and young children to improve schistosomiasis management of vulnerable populations. Responding to calls from the Federal Ministry of Economic Cooperation and Development of Germany (BMZ), CERMEL initiated a project on capacity building for fever management with focus on nonmalarial fever and antimicrobial resistance. This initiative led to the establishment of the national network for antimicrobial resistance in Gabon. Internationally, CERMEL became a key partner of the Organisation for Coordination and Cooperation in the Fight against Major Endemics in Central Africa (OCEAC) to control antimicrobial resistance in sub-Saharan Africa. CERMEL became an advisor of OCEAC for neglected tropical diseases.

## Clinical research in tuberculosis

Tuberculosis is one of the top causes of mortality worldwide, especially in HIV co-infected patients. To respond to more diverse and complicated cases of tuberculosis, a dedicated tuberculosis unit was established in 2009 at CERMEL. With support from the European Developing Countries Clinical Trial Partnership and the Bill and Melinda Gates Foundation, the German Center for Infection Research, and other funding bodies, the unit came to have a fully equipped tuberculosis diagnostic and research laboratory comprising smear microscopy, solid and liquid cultures and the full spectrum of molecular diagnostic and drug-resistant testing tools including GenXpert^TM^ (GenXpert, Cepheid, Sunnyvale, CA, US).

Continuous training of staff made it possible to sustain high-level laboratory services. In parallel, a comprehensive tuberculosis treatment consultancy system was established by a collaboration of clinicians and researchers at CERMEL. In collaboration with the National Tuberculosis Program the epidemiology of tuberculosis of the region was assessed [68]. This work promoted the understanding of the actual problem of multidrug-resistant tuberculosis (MDR-TB) in Gabon [69] and the findings led the government to act and improve the management of tuberculosis nationwide.

The MDR-TB ward was opened at Georges Rawiri Hospital (CHGR) in Lambaréné with the help of CERMEL to isolate patients and to provide training for nurses and physicians. Here, the so-called Bangladesh treatment regimen was assessed before it was officially recommended by the WHO as a standard of care. Based on these efforts, Gabon obtained for the first time support from the Global Fund for the national tuberculosis diagnosis and treatment program. Meanwhile, CERMEL became the national reference centre for tuberculosis diagnosis and later the primary recipient of the Global Fund program.

Tuberculosis treatment in the Moyen Ogooué Province around Lambaréné [70] and the socioanthropological determinants of tuberculosis control in Gabon [71] as well co-infections of HIV and tuberculosis were investigated [72]. These studies raised concerns for the quality of pediatric tuberculosis patients care [73] and the timely access to the second-line drugs for drug-resistant tuberculosis in Gabon [74]. To further improve patient adherence with treatment regimens a novel e‑Health tool is under development with support from the German Ministry for Economic Cooperation and Development.

## Clinical research on neglected tropical diseases

Clinical research on neglected tropical diseases (NTD) has been CERMEL’s research agenda since the 1990s with focus on *Schistosoma haematobium*, intestinal helminths, filarial and parasitic coinfections [75–79]. Studies assessing the burden of parasitic infections among pregnant women and children were performed [80–83]. Recently, a comprehensive disease burden of *Loa loa*, the African eye worm, was conducted to illustrate the clinical spectrum of the disease for the first time in Central Africa [84]. Interventional studies of repurposed drugs or alternative drug regimens for several neglected tropical diseases were performed, such as an assessment of the efficacy of albendazole to treat *Ascaris lumbricoides, Trichuris trichiura* and hookworm [85]. Similarly, mefloquine was evaluated as a potential alternative treatment for urogenital schistosomiasis in pregnant women [86]. Currently, innovative sequential treatment protocols for loiasis are being assessed. Additional studies investigating the influence of helminth infection on vaccine immunogenicity were conducted and supported by the Bill and Melinda Gates Foundation and the European Developing Countries Clinical Trial Partnership [60–62]. Projects to investigate the impact of helminth infections on pregnancy and birth outcomes are ongoing.

## Human infection models (CHIM) and insectarium

In addition to the classical observational studies and clinical trials, CERMEL started to employ controlled human infection models as new approach to infectious disease research. Here, healthy volunteers are deliberately exposed to an infectious agent to study the pathophysiology, immunology and transmission of human disease as well as to test novel preventive measures, such as vaccines and treatments. When the strict safety and ethical premises that are required for CHIM studies are met, a particular strength of the method lies in the definition of the timing and dose of inoculation, which enables clinical studies to be much smaller, shorter and thus more affordable than those by natural infection [87]. The use of CHIM is now well-established in medical sciences [87] to the extent that results of CHIM are now accepted in regular phase 3 clinical trials for regulatory processes [88]^1^.

In 2014, in part due to the active support from the Bill and Melinda Gates Foundation, the CERMEL became one of the first centres in Africa to perform CHIM. This study was to assess the effect of naturally acquired immunity and of sickle-cell trait on susceptibility to infection and disease [89]. This study demonstrated CERMEL’s capacity to stem the intensive logistical and clinical management, aseptically formulating an inoculum and providing continuous high-level diagnostic laboratory support. This led to follow-up studies using CHIM in malaria to assess efficacy of the malaria vaccine candidate GMZ2 [90] and whole cell immunization approaches (ongoing) by repeated infection of healthy adult Gabonese volunteers. The portfolio of CHIM has recently been extended to hookworm infections (*Necator americanus*).

While all CHIMs conducted at CERMEL in the past were done by injecting cryopreserved malaria parasites to humans, CHIM by insect (mosquito) bite, the natural way of inoculation, is also established. In 2017, the construction of a DZIF-funded bio safety level 3 (BSL-3) insectarium was completed, which houses state of the art facilities for maintaining mosquito colonies, cell cultures and a negative pressure area for safely maintaining and analyzing mosquitoes, and experimentally infecting them with BSL‑3 category pathogens (i.e. malaria, filariasis and arboviruses). The initial focus was on *P. falciparum* transmission but now also includes nonfalciparum malaria and viral pathogens.

## Fundamental research, clinical microbiology, and antimicrobial resistance research

CERMEL started basic research in the early 1990s, when the epidemiological landscape of the region became clearer. Facilities were continually upgraded and nowadays include laboratories for molecular biology, microbiology, cell culture and immunology as well as BSL‑3 laboratories. Due to an expansion of the microbiology facilities, culture-based methods (e.g. blood culture), antimicrobial susceptibility testing, genomic detection of resistance genes, serotyping and genotyping of bacteria have become possible to perform on site. Early scientific studies focused on the molecular epidemiology of bacterial pathogens, such as *Staphylococcus aureus*, extended spectrum beta-lactamase (ESBL)-producing *Enterobacterales, Streptococcus pneumoniae, Shigella spp*. and *Legionella spp*., and later newly discovered species, such as *Staphylococcus schweitzeri* [91–97].

CERMEL’s longstanding experience in clinical and fundamental research, the availability of adequate laboratory facilities including a dedicated BSL‑3 laboratory for applied medical virology and a considerable pool of trained scientists allowed CERMEL to rapidly adapt to the emergence of the severe acute respiratory syndrome coronavirus 2 (SARS-CoV-2) pandemic when it arrived in the Central African region. With the assistance of the international partners and emergency funding from German funding bodies, the CERMEL rapidly established a state of the art diagnostic laboratory service for molecular diagnostics of SARS-CoV‑2. In this way CERMEL served the public health service of the Republic of Gabon in its national response to the pandemic highlighting CERMEL’s dedication to serve the health needs of local populations and benefitting the public health system of Gabon.

## The health and demographic surveillance system (HDSS)

Since 2016, CERMEL hosts a health and demographic surveillance system in Lambaréné and surrounding villages. A total of 30,000 persons in 8300 households are followed up at least once per year. Verbal autopsies are done for all registered deaths. Data of the HDSS have been used as a sampling framework for various surveys including evidence-based estimates for pediatric vaccine coverage in rural Gabon.

## A hub for global health networks

A training program for graduate students in medicine and natural and social sciences was established at CERMEL in collaboration with domestic and overseas universities. The main partners are the Université Omar Bongo and Université des Sciences et Techniques de Masuku (Gabon), the Congolese Foundation for Medical Research (Republic of Congo), the University Clinics and the University of Tübingen and the Bernhard Nocht Institute for Tropical Medicine (Germany), the Medical University of Vienna (Austria), the Universities of Amsterdam and of Leiden (The Netherlands), and the St. George’s University of London (UK). Based on these academic networks several hundred students, medical doctors and scientists joined CERMEL for mid-term to long-term training or have been sent by CERMEL to these academic partner institutions for capacity building programs.

Over the past decade leading scientists of CERMEL were awarded professorships at prestigious international universities in Germany (University of Tübingen and Hamburg) and Austria (Medical University of Vienna). While being appointed as full professors at these international institutions, the place of work of these professors remains at CERMEL, thus avoiding a brain-drain for Gabon and intensifying the international collaborative network. A joint initiative with the Medical University of Vienna led to the appointment of a UNESCO professorship in bioethics with an office at the CERMEL. This initiative puts research ethics in the focus of all research programs at CERMEL and assists to further strengthen bioethical oversight of research in the Central African region [98].

A close collaboration with universities in Libreville and Franceville/Masuku, together with its recognition by African and Malagasy Council for Higher Education (CAMES) has made CERMEL a regional tertiary education centre. Through a cooperation with the Gabonese national programs for malaria, tuberculosis, HIV and neglected tropical diseases, CERMEL largely contributed to improve health care for the Gabonese population. As a founding member of the Central African Clinical Research Network (CANTAM)—a network of excellence funded by the European Developing Countries Clinical trial Partnership—CERMEL has increasing responsibilities in the research landscape of the Central African subregion. Firmly embedded in African and international academic networks, CERMEL thus became known as a reliable academic partner institution in the international landscape of clinical research [99].

## High-level infrastructures and sustainability of CERMEL

**Fig. 3:**
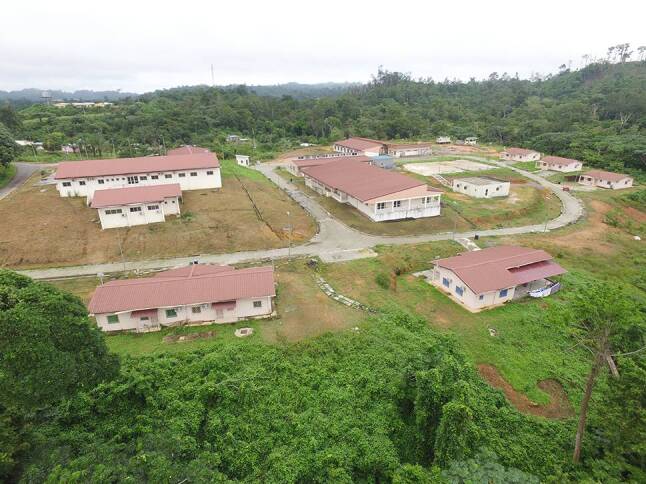
New research campus of the CERMEL with dedicated facilities for clinical research, entomology, and BSL‑4 virus research. (© Bertrand Lell)

In the last decade, CERMEL invested in the construction of advanced infrastructure to conduct clinical trials compliant with the guidelines of the International Council for Harmonisation of Technical Requirements for Pharmaceuticals for Human Use (ICH). The centre expanded from a single research building to a campus with more than ten buildings dedicated to specified clinical and research facilities and administrative departments (Fig. 3).

An institutional scientific review committee (SRC) was established to ensure safety, quality and adherence to legal requirements of the research projects to be conducted on site. An institutional ethics committee was also established for the CERMEL with the help of the European Developing Countries Clinical Trials Partnership to ensure that studies always adhere to the highest ethical standards. Following the establishment of these committees, CERMEL assisted with the support of the German Academic Exchange Service (DAAD) to establish the National Ethics Committee of Gabon.

## Conclusion

Starting as a research annex of a provincial rural hospital, CERMEL has been and will be committed to its philosophy to serve the local population in medicine, science and training. Medicine and global health research face continuous challenges, particularly so in Africa; however, medicine in Africa keeps on improving rapidly and due to the efforts of physicians, technical staff and scientists CERMEL is not a local research centre anymore. It became an international research centre headed by a board of directors and overseen by a dedicated board of trustees. Despite CERMEL’s inspiring development over the past decades, its leitmotiv to “improve medicine for local populations” has always remained the same.

## Footnotes

1 https://www.fda.gov/BiologicsBloodVaccines/Vaccines/ApprovedProducts/ucm505866.htm. https://www.fda.gov/downloads/BiologicsBloodVaccines/Vaccines/ApprovedProducts/UCM509681.pdf.

## References

[1] Burchard GD (1984). Plasmodium falciparum-malaria: resistance to chloroquine, but sensitivity to mefloquine in the Gabon. A prospective in-vitro study. Tropenmed Parasitol.

[2] Kremsner PG (1993). Curing of chloroquine-resistant malaria with clindamycin. Am J Trop Med Hyg.

[3] Kremsner PG (1994). Clindamycin in combination with chloroquine or quinine is an effective therapy for uncomplicated Plasmodium falciparum malaria in children from Gabon. J Infect Dis.

[4] Kremsner PG (1994). Comparison of micronized halofantrine with chloroquine-antibiotic combinations for treating Plasmodium falciparum malaria in adults from Gabon. Am J Trop Med Hyg.

[5] Metzger W (1995). High efficacy of short-term quinine-antibiotic combinations for treating adult malaria patients in an area in which malaria is hyperendemic. Antimicrob Agents Chemother.

[6] Metzger W (1995). Sulfadoxine/pyrimethamine or chloroquine/clindamycin treatment of Gabonese school children infected with chloroquine resistant malaria. J Antimicrob Chemother.

[7] Kremsner PG (1995). Quinine plus clindamycin improves chemotherapy of severe malaria in children. Antimicrob Agents Chemother.

[8] Radloff PD (1996). Atovaquone and proguanil for Plasmodium falciparum malaria. Lancet.

[9] Kremsner PG, Looareesuwan S, Chulay JD (1999). Atovaquone and proguanil hydrochloride for treatment of malaria. J Travel Med.

[10] Lell B (1998). Randomised placebo-controlled study of atovaquone plus proguanil for malaria prophylaxis in children. Lancet.

[11] Radloff PD (1996). Arteflene compared with mefloquine for treating Plasmodium falciparum malaria in children. Am J Trop Med Hyg.

[12] Schmidt-Ott R (1997). Pyrimethamine/sulfadoxine for treating uncomplicated Plasmodium falciparum malaria in young children in Gabon. Trans R Soc Trop Med Hyg.

[13] Adjuik M (2002). Amodiaquine-artesunate versus amodiaquine for uncomplicated Plasmodium falciparum malaria in African children: a randomised, multicentre trial. Lancet.

[14] Ramharter M (2005). Artesunate-clindamycin versus quinine-clindamycin in the treatment of Plasmodium falciparum malaria: a randomized controlled trial. Clin Infect Dis.

[15] Ramharter M (2008). Fixed-dose pyronaridine-artesunate combination for treatment of uncomplicated falciparum malaria in pediatric patients in Gabon. J Infect Dis.

[16] Borrmann S (2005). Short-course regimens of artesunate-fosmidomycin in treatment of uncomplicated Plasmodium falciparum malaria. Antimicrob Agents Chemother.

[17] The Four Artemisinin-Based Combinations (4ABC) Study Group (2011). A head-to-head comparison of four artemisinin-based combinations for treating uncomplicated malaria in African children: a randomized trial. PLoS Med.

[18] Lell B (2000). Malaria chemoprophylaxis with tafenoquine: a randomised study. Lancet.

[19] Han Z, Liang SY, Marschall J (2010). Current strategies for the prevention and management of central line-associated bloodstream infections. Infect Drug Resist.

[20] Missinou MA (2002). Fosmidomycin for malaria. Lancet.

[21] Lell B (2003). Fosmidomycin, a novel chemotherapeutic agent for malaria. Antimicrob Agents Chemother.

[22] Wiesner J, Borrmann S, Jomaa H (2003). Fosmidomycin for the treatment of malaria. Parasitol Res.

[23] Borrmann S (2004). Fosmidomycin-clindamycin for Plasmodium falciparum Infections in African children. J Infect Dis.

[24] Borrmann S (2004). Fosmidomycin-clindamycin for the treatment of Plasmodium falciparum malaria. J Infect Dis.

[25] Borrmann S (2004). Short report: evaluation of a simple and inexpensive photometric device for the measurement of hemoglobin. Am J Trop Med Hyg.

[26] Borrmann S (2006). Fosmidomycin plus clindamycin for treatment of pediatric patients aged 1 to 14 years with Plasmodium falciparum malaria. Antimicrob Agents Chemother.

[27] Oyakhirome S (2007). Randomized controlled trial of fosmidomycin-clindamycin versus sulfadoxine-pyrimethamine in the treatment of Plasmodium falciparum malaria. Antimicrob Agents Chemother.

[28] Mombo-Ngoma G (2018). Efficacy and safety of fosmidomycin-piperaquine as nonartemisinin-based combination therapy for uncomplicated falciparum malaria: a single-arm, age de-escalation proof-of-concept study in Gabon. Clin Infect Dis.

[29] Alloueche A (2004). Comparison of chlorproguanil-dapsone with sulfadoxine-pyrimethamine for the treatment of uncomplicated falciparum malaria in young African children: double-blind randomised controlled trial. Lancet.

[30] Held J (2015). Ferroquine and artesunate in African adults and children with Plasmodium falciparum malaria: a phase 2, multicentre, randomised, double-blind, dose-ranging, non-inferiority study. Lancet Infect Dis.

[31] Mombo-Ngoma G (2011). Phase I randomized dose-ascending placebo-controlled trials of ferroquine—a candidate anti-malarial drug—in adults with asymptomatic Plasmodium falciparum infection. Malar J.

[32] Supan C (2012). Pharmacokinetics of ferroquine, a novel 4-aminoquinoline, in asymptomatic carriers of Plasmodium falciparum infections. Antimicrob Agents Chemother.

[33] Macintyre F (2017). A randomised, double-blind clinical phase II trial of the efficacy, safety, tolerability and pharmacokinetics of a single dose combination treatment with artefenomel and piperaquine in adults and children with uncomplicated Plasmodium falciparum malaria. BMC Med.

[34] Mischlinger J, Agnandji ST, Ramharter M (2016). Single dose treatment of malaria—current status and perspectives. Expert Rev Anti Infect Ther.

[35] Kremsner PG (2012). A simplified intravenous artesunate regimen for severe malaria. J Infect Dis.

[36] Kremsner PG (2016). Intramuscular artesunate for severe malaria in African children: a multicenter randomized controlled trial. PLoS Med.

[37] Grobusch MP, Kremsner PG (2005). Uncomplicated malaria. Curr Top Microbiol Immunol.

[38] Adegnika AA (2005). Effectiveness of quinine monotherapy for the treatment of Plasmodium falciparum infection in pregnant women in Lambarene, Gabon. Am J Trop Med Hyg.

[39] Grobusch MP (2007). Intermittent preventive treatment against malaria in infants in Gabon—A randomized, double-blind, placebo-controlled trial. J Infect Dis.

[40] May J (2008). Therapeutic and prophylactic effect of intermittent preventive anti-malarial treatment in infants (IPTi) from Ghana and Gabon. Malar J.

[41] Aponte JJ (2009). Efficacy and safety of intermittent preventive treatment with sulfadoxine-pyrimethamine for malaria in African infants: a pooled analysis of six randomised, placebo-controlled trials. Lancet.

[42] González R (2014). Intermittent preventive treatment of malaria in pregnancy with mefloquine in HIV-negative women: a multicentre randomized controlled trial. PLoS Med.

[43] Ruperez M (2016). Mortality, morbidity, and developmental outcomes in infants born to women who received either mefloquine or sulfadoxine-pyrimethamine as intermittent preventive treatment of malaria in pregnancy: a cohort study. PLoS Med.

[44] Faucher JF (2002). Efficacy of atovaquone/proguanil for malaria prophylaxis in children and its effect on the immunogenicity of live oral typhoid and cholera vaccines. Clin Infect Dis.

[45] RTS,S Clinical Trials Partnership (2015). Efficacy and safety of RTS,S/AS01 malaria vaccine with or without a booster dose in infants and children in Africa: final results of a phase 3, individually randomised, controlled trial. Lancet.

[46] Mordmüller B (2010). Safety and immunogenicity of the malaria vaccine candidate GMZ2 in malaria-exposed, adult individuals from Lambaréné, Gabon. Vaccine.

[47] Mordmüller B (2019). First-in-human, randomized, double-blind clinical trial of differentially adjuvanted PAMVAC, a vaccine candidate to prevent pregnancy-associated malaria. Clin Infect Dis.

[48] Dejon-Agobe JC (2019). Controlled human malaria infection of healthy adults with lifelong malaria exposure to assess safety, Immunogenicity, and efficacy of the asexual blood stage malaria vaccine candidate GMZ2. Clin Infect Dis.

[49] Bélard S (2011). A randomized controlled phase Ib trial of the malaria vaccine candidate GMZ2 in African children. PLoS ONE.

[50] Poetsch JH (2019). Detectable Vesicular Stomatitis virus (VSV)-specific humoral and cellular immune responses following VSV-Ebola virus vaccination in humans. J Infect Dis.

[51] Huttner A (2018). Determinants of antibody persistence across doses and continents after single-dose rVSV-ZEBOV vaccination for Ebola virus disease: an observational cohort study. Lancet Infect Dis.

[52] Agnandji ST (2017). Safety and immunogenicity of rVSV∆G-ZEBOV-GP Ebola vaccine in adults and children in Lambaréné, Gabon: a phase I randomised trial. PLoS Med.

[53] Rechtien A (2017). Systems vaccinology identifies an early innate immune signature as a correlate of antibody responses to the Ebola vaccine rVSV-ZEBOV. Cell Rep.

[54] Dahlke C (2017). Dose-dependent T-cell dynamics and cytokine cascade following rVSV-ZEBOV immunization. EBioMedicine.

[55] Huttner A (2017). A dose-dependent plasma signature of the safety and immunogenicity of the rVSV-ebola vaccine in Europe and Africa. Sci Transl Med.

[56] Medaglini D (2015). Ebola vaccine R&D: Filling the knowledge gaps. Sci Transl Med.

[57] Agnandji ST (2016). Phase 1 trials of rVSV ebola vaccine in Africa and Europe. N Engl J Med.

[58] van Riet E (2007). Cellular and humoral responses to influenza in gabonese children living in rural and semi-urban areas. J Infect Dis.

[59] van Riet E (2008). Cellular and humoral responses to tetanus vaccination in Gabonese children. Vaccine.

[60] Esen M (2012). Reduced antibody responses against Plasmodium falciparum vaccine candidate antigens in the presence of Trichuris trichiura. Vaccine.

[61] Brückner S (2015). Effect of antihelminthic treatment on vaccine immunogenicity to a seasonal influenza vaccine in primary school children in Gabon: a randomized placebo-controlled trial. PLoS Negl Trop Dis.

[62] Brückner S (2016). A single-dose antihelminthic treatment does not influence immunogenicity of a meningococcal and a cholera vaccine in Gabonese school children. Vaccine.

[63] Planche T (2001). Comparison of methods for the rapid laboratory assessment of children with malaria. Am J Trop Med Hyg.

[64] Taylor T (2006). Standardized data collection for multi-center clinical studies of severe malaria in African children: establishing the SMAC network. Trans R Soc Trop Med Hyg.

[65] Kendjo E (2013). Mortality patterns and site heterogeneity of severe malaria in African children. PLoS ONE.

[66] Kremsner PG (2009). Prognostic value of circulating pigmented cells in African children with malaria. J Infect Dis.

[67] Helbok R (2009). The Lambaréné Organ Dysfunction Score (LODS) is a simple clinical predictor of fatal malaria in African children. J Infect Dis.

[68] Bélard S (2016). Tuberculosis treatment outcome and drug resistance in Lambaréné, Gabon: a prospective cohort study. Am J Trop Med Hyg.

[69] Alabi AS (2017). Enhanced laboratory capacity development: a boost for effective tuberculosis control in resource-limited settings. Int J Infect Dis.

[70] Stolp SM (2013). Tuberculosis patients hospitalized in the Albert Schweitzer Hospital, Lambaréné, Gabon—A retrospective observational study. Clin Microbiol Infect.

[71] Cremers AL (2013). Perceptions, health care seeking behaviour and implementation of a tuberculosis control programme in Lambaréné, Gabon. Public Health Action.

[72] Janssen S (2014). TB and HIV in the Central African region: current knowledge and knowledge gaps. Infection.

[73] Flamen A (2014). Childhood tuberculosis in Lambaréné, Gabon: tuberculosis control in its infancy?. Infection.

[74] Bélard S (2015). Limited access to drugs for resistant tuberculosis: a call to action. J Public Health (Oxf).

[75] Grogan JL (1996). Antischistosome IgG4 and IgE responses are affected differentially by chemotherapy in children versus adults. J Infect Dis.

[76] Van Etten L (1997). Day-to-day variation of egg output and schistosome circulating antigens in urine of Schistosoma haematobium-infected school children from Gabon and follow-up after chemotherapy. Am J Trop Med Hyg.

[77] van den Biggelaar AH (2000). Decreased atopy in children infected with Schistosoma haematobium: a role for parasite-induced interleukin-10. Lancet.

[78] Borrmann S (2001). Artesunate and praziquantel for the treatment of Schistosoma haematobium infections: a double-blind, randomized, placebo-controlled study. J Infect Dis.

[79] van der Kleij D (2004). Responses to Toll-like receptor ligands in children living in areas where schistosome infections are endemic. J Infect Dis.

[80] Dejon-Agobe JC (2018). Schistosoma haematobium effects on Plasmodium falciparum infection modified by soil-transmitted helminths in school-age children living in rural areas of Gabon. PLoS Negl Trop Dis.

[81] Mombo-Ngoma G (2017). Urogenital schistosomiasis during pregnancy is associated with low birth weight delivery: analysis of a prospective cohort of pregnant women and their offspring in Gabon. Int J Parasitol.

[82] Mombo-Ngoma G (2015). Loa loa infection in pregnant women, Gabon. Emerg Infect Dis.

[83] Adegnika AA (2010). Epidemiology of parasitic co-infections during pregnancy in Lambarene, Gabon. Trop Med Int Health.

[84] Veletzky L, Hergeth J, Stelzl DR, Mischlinger J, Manego RZ, Mombo-Ngoma G, McCall MBB, Adegnika AA, Agnandji ST, Metzger WG, Matsiegui PB, Lagler H, Mordmüller B, Budke C, Ramharter M (2020). Burden of disease in Gabon caused by loiasis: a cross-sectional survey. Lancet Infect Dis.

[85] Adegnika AA (2014). Randomized, controlled, assessor-blind clinical trial to assess the efficacy of single- versus repeated-dose albendazole to treat ascaris lumbricoides, trichuris trichiura, and hookworm infection. Antimicrob Agents Chemother.

[86] Basra A (2013). Efficacy of mefloquine intermittent preventive treatment in pregnancy against Schistosoma haematobium infection in Gabon: a nested randomized controlled assessor-blinded clinical trial. Clin Infect Dis.

[87] Roestenberg M (2018). Experimental infection of human volunteers. Lancet Infect Dis.

[88] Chen WH (2016). Single-dose live oral cholera vaccine CVD 103-HgR protects against human experimental infection with Vibrio cholerae O1 el Tor. Clin Infect Dis.

[89] Lell B (2018). Impact of sickle cell trait and naturally acquired immunity on uncomplicated malaria after controlled human malaria infection in adults in Gabon. Am J Trop Med Hyg.

[90] Dejon-Agobe JC (2018). Controlled human malaria infection of healthy lifelong malaria-exposed adults to assess safety, immunogenicity and efficacy of the asexual blood stage malaria vaccine candidate GMZ2. Clin Infect Dis.

[91] Tong SYC (2015). Novel staphylococcal species that form part of a Staphylococcus aureus-related complex: the non-pigmented Staphylococcus argenteus sp. nov. and the non-human primate-associated Staphylococcus schweitzeri sp. nov. Int J Syst Evol Microbiol.

[92] Ateba Ngoa U (2012). Epidemiology and population structure of Staphylococcus aureus in various population groups from a rural and semi urban area in Gabon, Central Africa. Acta Trop.

[93] Herrmann M (2013). Staphylococcal disease in Africa: another neglected ‘tropical’ disease. Future Microbiol.

[94] Alabi AS (2013). Retrospective analysis of antimicrobial resistance and bacterial spectrum of infection in Gabon, Central Africa. BMC Infect Dis.

[95] Schaumburg F (2011). Virulence factors and genotypes of Staphylococcus aureus from infection and carriage in Gabon. Clin Microbiol Infect.

[96] Ehrhardt J (2013). Population structure of Legionella spp. from environmental samples in Gabon. Infect Genet Evol.

[97] Schaumburg F (2013). Streptococcus pneumoniae colonization in remote African Pygmies. Trans R Soc Trop Med Hyg.

[98] Druml C (2016). Bioethics internationally and in Austria: a sense of solidarity. Wien Klin Wochenschr.

[99] Miiro GM (2013). EDCTP regional networks of excellence: initial merits for planned clinical trials in Africa. BMC Public Health.

